# Probing the Effects and Mechanisms of Electroacupuncture at Ipsilateral or Contralateral ST36–ST37 Acupoints on CFA-induced Inflammatory Pain

**DOI:** 10.1038/srep22123

**Published:** 2016-02-24

**Authors:** Kung-Wen Lu, Chao-Kuei Hsu, Ching-Liang Hsieh, Jun Yang, Yi-Wen Lin

**Affiliations:** 1 College of Chinese Medicine, School of Post-Baccalaureate Chinese Medicine, China Medical University, Taichung 40402, Taiwan; 2 China Medical University Hospital, Department of Chinese Medicine, Taichung 40402, Taiwan; 3 College of Chinese Medicine, Graduate Institute of Acupuncture Science, China Medical University, Taichung 40402, Taiwan; 4 Taichung Armed forces General Hospital, Department of Orthopedics, Taichung 41152, Taiwan; 5 National Defense Medical Center, Taipei 114, Taiwan; 6 College of Chinese Medicine, Graduate Institute of Integrative Medicine, China Medical University, Taichung 40402, Taiwan; 7 Research Center for Chinese Medicine & Acupuncture, China Medical University, Taichung 40402, Taiwan

## Abstract

Transient receptor potential vanilloid 1 (TRPV1) and associated signaling pathways have been reported to be increased in inflammatory pain signaling. There are accumulating evidences surrounding the therapeutic effect of electroacupuncture (EA). EA can reliably attenuate the increase of TRPV1 in mouse inflammatory pain models with unclear signaling mechanisms. Moreover, the difference in the clinical therapeutic effects between using the contralateral and ipsilateral acupoints has been rarely studied. We found that inflammatory pain, which was induced by injecting the complete Freund’s adjuvant (CFA), (2.14 ± 0.1, *p* < 0.05, n = 8) can be alleviated after EA treatment at either ipsilateral (3.91 ± 0.21, *p* < 0.05, n = 8) or contralateral acupoints (3.79 ± 0.25, *p* < 0.05, n = 8). EA may also reduce nociceptive Nav sodium currents in dorsal root ganglion (DRG) neurons. The expression of TRPV1 and associated signaling pathways notably increased after the CFA injection; this expression can be further attenuated significantly in EA treatment. TRPV1 and associated signaling pathways can be prevented in TRPV1 knockout mice, suggesting that TRPV1 knockout mice are resistant to inflammatory pain. Through this study, we have increased the understanding of the mechanism that both ipsilateral and contralateral EA might alter TRPV1 and associated signaling pathways to reduce inflammatory pain.

Inflammatory pain may result from thermal, chemical, or mechanical injuries that conduct signal transmission through nociceptors in the nervous system^1^. An inflammatory state can be initiated by injecting chemical agents such as the complete Freund’s adjuvant (CFA) or carrageenan^2^,^3^,^4^. Inflammation could activate both mechanical and thermal transducers such as transient receptor potential vanilloid 1 (TRPV1)^5^,^6^, acid-sensing ion channels (ASICs)^7^, TRPV4^8^, TRPA1^9^, and TREK1/2^10^. Both local and systemic inflammation decrease pain threshold^11^,^12^.

TRPV1 is a ion channel that is expressed in nociceptive neurons in the dorsal root ganglion (DRG), dorsal horn of the spinal cord (SCDH), the brain, and peripheral tissue^3^,^13^. TRPV1 is activated by acidic conditions, capsaicin, and heat (temperature > 43 °C)^13^,^14^, and activated TRPV1 can lead to mechanical or thermal hyperalgesia^15^,^16^. Alternatively, blocking TRPV1 can attenuate thermal hyperalgesia^17^. In a recent study, the authors found that TRPV1 played an important role in neuropathic pain^18^. Recent study found that thermal and mechanical hyperalgesia caused by CFA-induced inflammation led to increased TRPV1 expression in DRG neurons for 28 days^19^. Interestingly, the mechanism of TRPV1 is not as simple as described above. TRPV1 has been shown to respond to different stimuli depending on where the channel is located; peripherally expressed TRPV1 is involved in thermal hyperalgesia, whereas centrally expressed TRPV1 is involved in detecting both thermal and mechanical pain^20^,^21^,^22^. TRPV1 is highly expressed in the DRG^2^ and also expressed in brain regions such as the cortex, cerebellum, hippocampus, and others area^23^. TRPV1 is also reported important in inflammatory pain and associated with pain-related molecules such as PKA^24^, PI3K^25^, and PKC^26^.

Contralateral needling has been used to treat disease in traditional Chinese medicine for a long time. Koo *et al.* created an ankle sprain animal model, wherein the contralateral forelimbs could be treated using the endogenous opioid system^27^. A subsequent study indicated that the analgesic effect was mediated by the spinal α2-adrenoceptor^28^. Somers *et al.* used contralateral transcutaneous electrical nerve stimulation (TENS) in rats with chronic constriction (CCI) and found improvements in mechanical, but not thermal, allodynia^29^. Yang *et al.* studied contralateral acupuncture in 2011, and they found that low frequency EA could reduce hyperalgesia induced by carrageenan and that the mechanism for the pain reduction used μ-opioid receptors in the SC^30^.

Acupuncture is a useful method for treating pain, and it has been used for a long time in Asia. Ancient books describe a variety of acupuncture techniques and different theories of pain management, including contralateral acupuncture. Due to its effectiveness in analgesia, people have investigated the mechanisms of pain relief acupuncture. In 1981, Melzack proposed the gate control theory to explain the analgesic effect of needling in trigger points^31^. Later the endorphin theory was proposed, which provided a stronger scientific evidence^32^. There are many studies about the analgesic mechanisms of acupuncture, which suggests that many factors are involved in the nociceptive pathway, including descending noradrenergic and serotonergic pathways^33^,^34^. In recent studies, it was found that EA might inhibit the release of proinflammatory cytokines such as IL-1, IL-6, TNF-α, and p38^35^. Recent article indicated that EA could reduce the expression of TRPV1 in a mice fibromyalgia pain model^36^.

Although theories have been proposed and studies have been conducted about contralateral acupuncture, they did not mention the difference between using ipsilateral and contralateral acupoints. The purpose of this study was to investigate whether there was a difference in analgesic effect following acupuncture at an ipsilateral acupoint compared to a contralateral acupoint. We did EA treatment at either the ipsilateral or contralateral side, and we found that they share similar mechanisms. We found that EA can reduce inflammatory pain induced by CFA and also alleviate nociceptive Nav sodium currents. EA also attenuated the overexpression of TRPV1 and associated signaling pathways that were increased after CFA injection. The potentiation of TRPV1 and associated pathways could be further avoided by deleting TRPV1 gene. Aforementioned mechanisms might be crucial and clear methods pertaining to EA analgesia.

## Materials and Methods

### Experimental Animals

Experiments were carried out on C57/B6 mice (aged 8 to 12 weeks) purchased from BioLASCO Co., Ltd, Taipei, Taiwan. After arrival, 12hr light-dark cycle with sufficient water and food were given. All procedures were approved by the Institute of Animal Care and Use Committee of China Medical University (permit No. 101-116-N) and were in accordance with *Guide for the use of Laboratory Animals* by National Research Council and with the ethical guideline of the International Association for the study of pain. The number of animal used and their suffering were minimized.

#### Inflammatory Pain Model and Behavior Test

Mice were anesthetized with 1–2% isoflurane and administered a injection of 20 μl saline (pH 7.4, buffered with 20 mM HEPES) or CFA (complete Freund’s adjuvant; 0.5 mg/ml heat-killed M. tuberculosis [Sigma, St. Louis, MO]) in the plantar surface of the hind paw to induce intraplantar inflammation. Behavior tests were conducted at 1–4 day after induction of inflammation, and DRGs were harvested on day 4. All experiments were performed at room temperature (approximately 25 °C) and the stimuli were applied only when the animals were calm but not sleeping or grooming. Mechanical sensitivity was measured by testing the force of responses to stimulation with three applications of electronic von Frey filaments (North Coast Medical, Gilroy, CA, USA). Thermal pain was measured with three applications using Hargraves’ test IITC analgesiometer (IITC Life Sciences, SERIES8, Model 390 G).

#### Electroacupuncture

There were four groups in this study: Control group; CFA group; ipsilateral group, EA was done at the same (right) side of CFA-induced inflammation, while the contralateral group was at left side. EA was done at ST36 and ST37 acupoints with stainless steel acupuncture needles (0.5 inch, 30 G, Yu-Kuang, Taiwan). We compared the therapeutic effect between ipsilateral and contralateral EA without sham control because it was ineffective^2^,^4^,^36^. The needles were inserted in 2–3 mm depth muscle layer at the acupoints. Electrical stimulation was produced by Trio 300 electrical stimulator (Grand Medical Instrument CO., LTD). The duration of each EA was 15 minutes; the setting of EA was 2 Hz in frequency, 1 mA in stimulation amplitude. EA was performed 30 min after CFA injection in day 1, in the morning (9:00 am–10:00 am) in day 2 and day 4. The procedure was done under anesthesia with 1% isoflurane in room temperature (25°C). The control group without EA treatment was also under anesthesia condition.

#### Tissue sampling and Western blotting

We dissected out the dorsal aspect of the vertebral column using scissors and forceps. The spinal cord was further lifted out and dorsal horn was collected by scissors. L3-L5 DRG and SCDH neurons were immediately excised to extract proteins. Total proteins were prepared by homogenized DRG and SCDH in lysis buffer containing 50 mM Tris-HCl pH 7.4, 250 mM NaCl, 1% NP−40, 5 mM EDTA, 50 mM NaF, 1 mM Na3VO4, 0.02% NaN3 and 1 × protease inhibitor cocktail (AMRESCO). The extracted proteins (30 μg per sample assessed by BCA protein assay) were subjected to 8% SDS-Tris glycine gel electrophoresis and transferred to a PVDF membrane. The membrane was blocked with 5% nonfat milk in TBS-T buffer (10 mM Tris pH 7.5, 100 mM NaCl, 0.1% Tween 20), incubated with first antibody in TBS-T with 1% bovine serum albumin, and incubated for 1 hour at room temperature. Peroxidase-conjugated anti-rabbit antibody (1:5000) was used as a secondary antibody. The bands were visualized by an enhanced chemiluminescencent substrate kit (PIERCE) with LAS-3000 Fujifilm (Fuji Photo Film Co. Ltd). Where applicable, the image intensities of specific bands were quantified with NIH ImageJ software (Bethesda, MD, USA).

### DRG primary cultures and Whole-cell patch-clamp recording

Mice aged 8–12 weeks were killed by use of CO_2_ to minimize their suffering. Lumbar (L3–L5) DRG neurons were dissected from ipsilateral site and placed in a tube containing DMEM and then transferred to DMEM with type I collagenase (0.125%, 120 min) for digestion at incubator at 37 °C. Neurons were then plated on poly-L-lysine-coated cover slides. All recordings were completed within 24 hours after plating. Glass pipettes (Warner Products 64–0792) were prepared (3–5 MΩ) with use of a vertical puller (NARISHIGE PC-10). Whole-cell recordings involved use of an Axopatch MultiClamp 700B (Axon Instruments). Stimuli were controlled and digital records captured with use of Signal 3.0 software and a CED1401 converter (Cambridge Electronic Design). Cells with a membrane potential more positive than −40 mV were not accepted. The bridge was balanced in current clamping recording and series resistance was compensated 70% in voltage-clamping recording with Axopatch 700B compensation circuitry. Recording cells were superfused in artificial cerebrospinal fluid (ACSF) containing (in mM) 130 NaCl, 5 KCl, 1 MgCl2, 2 CaCl2, 10 glucose, and 20 HEPES, adjusted to pH 7.4 with NaOH. ACSF solutions were applied by use of gravity. The recording electrodes were filled with (in mM) 100 KCl, 2 Na2-ATP, 0.3 Na3-GTP, 10 EGTA, 5 MgCl2, and 40 HEPES, adjusted to pH 7.4 with KOH. Osmolarity was approximately 300–310 mOsm. Capsaicin was prepared from a 100-μM stock solution (in 100% ethanol) to a final concentration of 1 μM in ACSF. All drugs were purchased from Sigma Chemical (St. Louis, MO, USA).

#### Statistical Analysis

All statistic data are presented as the mean ± standard error. Statistical significance between control, inflammation, and EA group was tested using the ANOVA test, followed by a post hoc Tukey’s test (*p* < 0.05 was considered statistically significant).

## Results

### Mechanical and thermal hyperalgesia induced by CFA injection was suppressed by both ipsilateral and contralateral EA treatments

**Figure 1:**
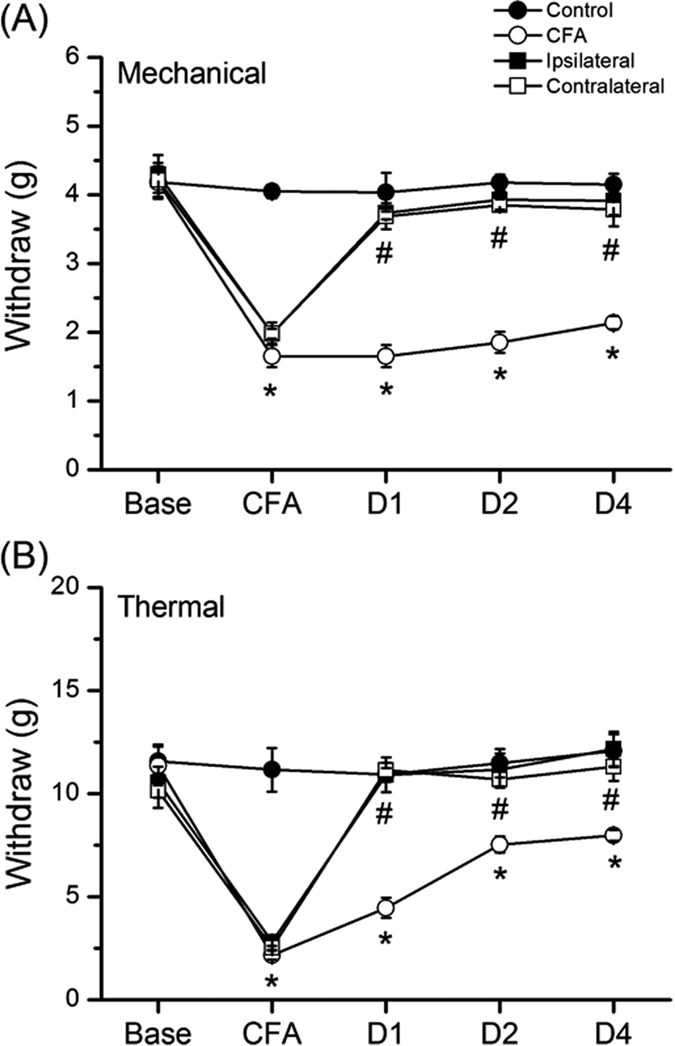
Changes in the withdraw threshold and latency of mice in the von Frey and radial heat test. The picture shows that analgesic effect of EA could be detected immediately (30 min) after treatment. There was no statistically significant difference in following EA treatments at either right or left acupoints. (Control: saline injection; CFA: CFA injection; ipsilateral: EA treatment on the same side of CFA-induced inflammation; contralateral: EA treatment on the contralateral side of inflammation. *p < 0.05 as compared with control group, ^#^*p* < 0.05 as compared with CFA group, n = 8.

To test whether ipsilateral or contralateral EA could equally reverse CFA-induced mechanical hyperalgesia, we compared responses to electric von Frey filaments at different days among control, CFA, ipsilateral, and contralateral EA groups. An injection of normal saline did not induce mechanical hyperalgesia (Fig. 1A, 4.15 ± 0.16 g, *p* < 0.05, compared with control group, n = 8), whereas an injection of CFA induced mechanical hyperalgesia in the hindpaw of mice on day 4 (Fig. 1A, 2.14 ± 0.1 g, *p* < 0.05, compared with control group, n = 8). The mechanical hyperalgesia was attenuated following either ipsilateral (Fig. 1A, 3.91 ± 0.21 g, *p* < 0.05, compared with CFA group, n = 8) or contralateral EA (Fig. 1A, 3.79 ± 0.25 g, *p* < 0.05, compared with CFA group, n = 8). Next, we utilized radial heat latency to define the degree of thermal hyperalgesia in mice. An injection of normal saline did not induce thermal hyperalgesia (Fig. 1B, 12.08 ± 0.79 s, *p* < 0.05, compared with control group, n = 8), whereas an injection of CFA induced thermal hyperalgesia on day 4 (Fig. 1B, 7.98 ± 0.25 s, *p* < 0.05, compared with control group, n = 8). However, the thermal hyperalgesia was abolished following either ipsilateral (Fig. 1B, 12.15 ± 0.83 s, *p* < 0.05, compared with CFA group, n = 8) or contralateral EA (Fig. 1B, 11.3 ± 0.68 s, *p* < 0.05, compared with CFA group, n = 8). These results demonstrated that EA at either the ipsilateral or the contralateral site of the ST36 acupoint could alleviate both mechanical and thermal hyperalgesia in CFA-induced inflammatory pain models.

### Nav sodium currents were increased in CFA mice and attenuated by EA in DRG neurons

**Figure 2:**
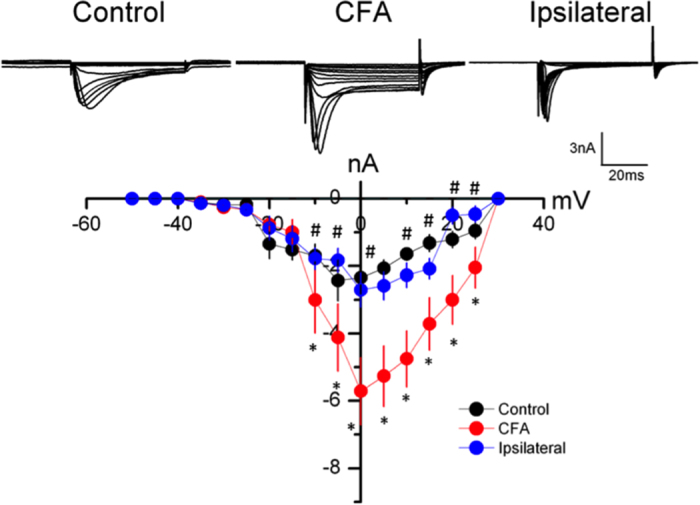
Nav sodium currents in L3–L5 DRG neurons. (**A**) Representative Nav current traces following step depolarizations in Con, CFA, and EA groups. The Nav currents were induced by membrane potential depolarizations from −50 to +30 mV. (**B**) Mean peak amplitudes of Nav currents in each group. ^∗^*p* < 0.05 compared to control group. ^#^*p* < 0.05 compared to CFA group.

To investigate whether the Nav sodium currents can be regulated by EA during CFA-induced inflammatory pain, we used whole-cell patch recordings to measure these currents. We depolarized DRG neurons from −50 to +30 mV to induce the currents. In the control group, the currents existed in DRG neurons and potentiated at 4 days after intraplantar CFA-induced inflammation (Fig. 2, *p* < 0.05, compared with control group, n = 8). Furthermore, EA significantly alleviated the increased Nav sodium currents, suggesting that the effect of the currents was reversible (Fig. 2, *p* < 0.05, compared with CFA group, n = 8).

### Activation of TRPV1 with capsaicin dramatically increased the amplitude of Nav sodium currents

**Figure 3:**
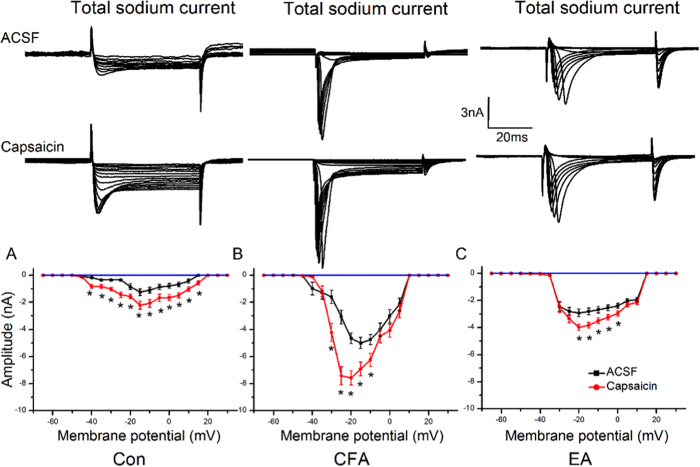
Nav sodium currents were modulated by TRPV1 activation in DRG neurons. (**A**) Representative Nav current traces in Con, CFA, and EA groups. The Nav currents were induced by membrane potential depolarizations to from −50 to + 30 mV. (**B**) Mean peak amplitudes of Nav currents were potentiated by capsaicin, a TRPV1 agonist, in each group. ^∗^*p* < 0.05compared to control group. ^#^*p* < 0.05 compared to CFA group.

To test whether TRPV1 activation can reliably enhance Nav sodium currents, we applied the TRPV1 agonist capsaicin directly to DRG neurons after the induction of Nav currents. We found that capsaicin increased Nav currents in control DRG neurons (Fig. 3A, *p* < 0.05, compared with control group, n = 8). Next, we added capsaicin to inflamed DRG neurons, and we found that TRPV1 significantly potentiated the Nav currents (Fig. 3B, *p* < 0.05, compared with control group, n = 8). Furthermore, similar results were also obtained in DRG neurons from EA mice, suggesting its role in enhancing Nav currents (Fig. 3C, *p* < 0.05, compared with control group, n = 8). Together, these results indicated that activation of TRPV1 could enhance Nav currents in DRG neurons.

### Expression of TPRV1 and pain-associated molecules in DRG neurons

**Figure 4:**
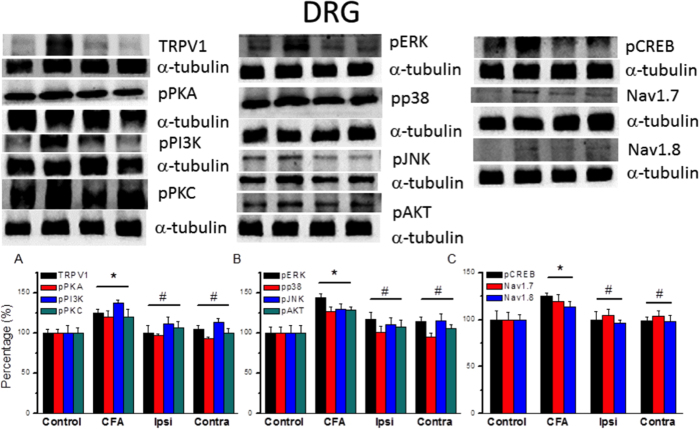
Expression levels of TRPV1 and associated signaling pathway protein in L3–5 DRG after CFA inflammation and EA treatment. (**A–C**) Proportions of immunopositive neurons. (**A**) TRPV1, pPKA, pPI3K, and pPKC expression in tissue from control, CFA, ipsilateral, and contralateral EA mice (from left to right). (**B**) Expression of pERK, pp38, pJNK, and pAKT. (**C**) Expression of pCREB, Nav1.7, and Nav1.8. α-tubulin was used as the internal control. Con = Control; CFA = CFA-induced inflammatory pain; Ipsi = electroacupuncture at ipsilateral site. Contra = electroacupuncture at contralateral site. ^∗^*p* < 0.05 compared to control group. ^#^*p* < 0.05 compared to CFA group.

Next, we used western blotting to measure the expression levels of TRPV1 and pain-associated molecules following a CFA injection and/or the EA manipulation. TRPV1-immunoreactive (IR) cells were distributed in the DRG. The density of TRPV1-IR was higher in the CFA group than in the control group on day 4 (Fig. 4A, 125.16 ± 4.85, *p* < 0.05, compared with control group, n = 6) and was at baseline in the CFA + EA group (Fig. 4A, Ipsilateral: 99.62 ± 9.94; Contralateral: 104.76 ± 4.97, *p* < 0.05, compared with CFA group, n = 6). Similar to TRPV1 expression, pPKA (Fig. 4A, 120.35 ± 7.67, *p* < 0.05, compared with control group, n = 6), pPI3K (Fig. 4A, 137.38 ± 3.35, *p* < 0.05, compared with control group, n = 6), and pPKC (Fig. 4A, 120.53 ± 9.12, *p* < 0.05, compared with control group, n = 6) were potentiated after CFA-induced inflammatory pain and further attenuated by EA stimulation. Next, we examined downstream mechanisms of phosphorylated protein kinases, our results indicated pERK (Fig. 4B, 144.21 ± 4.67, *p* < 0.05, compared with control group, n = 6), pp38 (Fig. 4B, 126.68 ± 5.79, *p* < 0.05, compared with control group, n = 6), pJNK (Fig. 4B, 129.53 ± 7.12, *p* < 0.05, compared with control group, n = 6), and pAKT (Fig. 4B, 128.4 ± 4.33, *p* < 0.05, compared with control group, n = 6) were all increased by CFA injection and can be further attenuated by EA. Next we found that pCREB was increased following the CFA injection (Fig. 4C, 124.97 ± 3.73, *p* < 0.05, compared with control group, n = 6) and reversed by EA, suggesting a transcriptional pathway for EA analgesia. We further determined that Nav1.7 (Fig. 4C, 118.98 ± 7.87, *p* < 0.05, compared with control group, n = 6) and Nav1.8 (Fig. 4C, 113.72 ± 5.27, *p* < 0.05, compared with control group, n = 6) were increased in CFA mice and further alleviated by EA. These results suggest that the upregulation of TRPV1 and associated signaling pathways may contribute to inflammatory hyperalgesia, whereas the reversal of this upregulation may account for the EA analgesia.

### Expression levels of TPRV1-associated molecules in SC neurons

**Figure 5:**
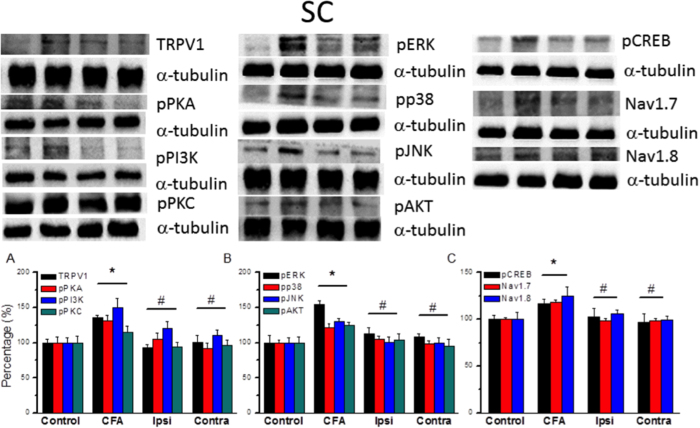
Expression levels of TRPV1 and associated signaling pathway proteins in SCDH after CFA inflammation and EA treatment. (**A–C**) Proportions of immunopositive neurons. (**A**) TRPV1, pPKA, pPI3K, and pPKC expression in Con, CFA, Ipsi, and Contra EA groups (**B**) Expression of pERK, pp38, pJNK, and pAKT. (**C**) Expression of pCREB, Nav1.7, and Nav1.8. α-tubulin was used as the internal control. α-tubulin was used as the internal control. Con = Control; CFA = CFA induced inflammatory pain; Ipsi = electroacupuncture at ipsilateral site; Contra = electroacupuncture at contralateral site. ^∗^*p* < 0.05 compared to control group. ^#^*p* < 0.05 compared to CFA group.

The expression of TPRV1 and associated molecules was also detected in the SC. Compared with the control group, the percentage of TRPV1 protein expression was increased in the CFA group (Fig. 5A, 135.36 ± 4.05, *p* < 0.05, compared with control group, n = 6) and reduced in two EA groups. Similar to the results in the DRG, the expression of pPKA (Fig. 5A, 130.73 ± 8.31, *p* < 0.05, compared with control group, n = 6), pPI3K (Fig. 5A, 150.12 ± 13.43, *p* < 0.05, compared with control group, n = 6), and pPKC (Fig. 5A, 114.92 ± 8.88, *p* < 0.05, compared with control group, n = 6) was increased after the CFA manipulation and further reduced by EA stimulation. Furthermore, we found that the expression of pERK (Fig. 5B, 154.75 ± 5.5, *p* < 0.05, compared with control group, n = 6), pp38 (Fig. 5B, 121.87 ± 4.77, *p* < 0.05, compared with control group, n = 6), pJNK (Fig. 5B, 130.36 ± 4.13, *p* < 0.05, compared with control group, n = 6), and pAKT (Fig. 5B, 124.95 ± 4.53, *p* < 0.05, compared with control group, n = 6) was also increased following the CFA injection and can be further ameliorated by EA in SC. Moreover, the expression of pCREB (Fig. 5C, 116.84 ± 4.46, *p* < 0.05, compared with control group, n = 6), Nav1.7 (Fig. 5C, 118.05 ± 2.47, *p* < 0.05, compared with control group, n = 6), and Nav1.8 (Fig. 5A, 124.54 ± 10.44, *p* < 0.05, compared with control group, n = 6) was also increased by CFA injection and reversed by EA. Taken together, these results suggest that the upregulation of TRPV1 and associated signal mechanisms may contribute to inflammatory hyperalgesia in the central SCDH.

### Overexpression of TRPV1 and associated signaling pathways were abolished in TRPV1^−/−^ mice DRG and SCDH

**Figure 6:**
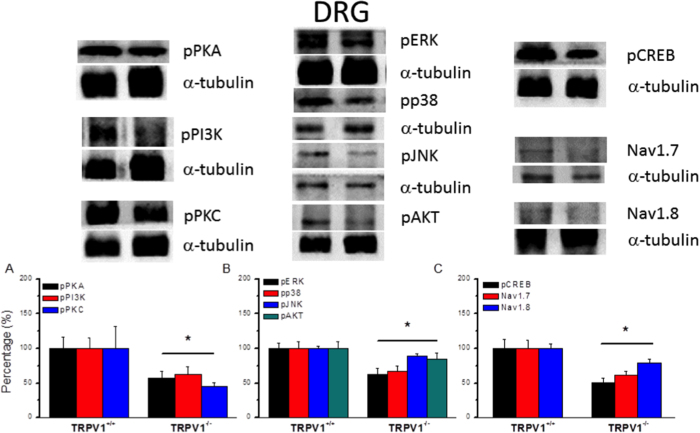
Expression levels of TRPV1 associated signaling pathways in L3–L5 DRG from TRPV1 null mice. (**A**) Western blots of DRG lysates probed for pPKA, pPI3K, and pPKC. (**B**) Expression levels of pERK, pp38, pJNK, and pAKT. (**A**) Western blots of DRG lysates probed for pCREB, Nav1.7, and Nav1.8. α-tubulin was the internal control. *p < 0.05.

**Figure 7:**
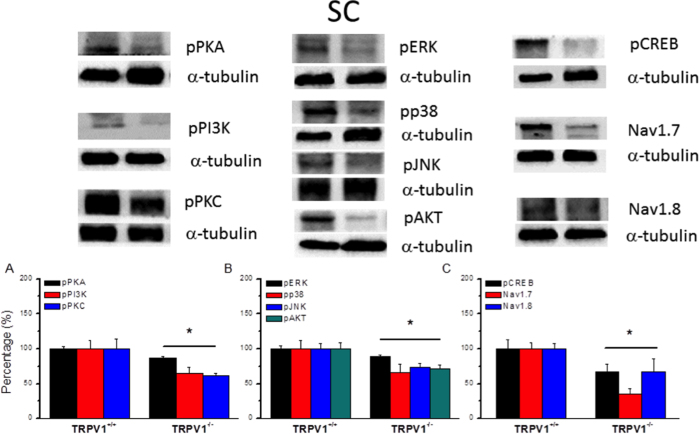
Expression levels of TRPV1 associated signaling pathways in SC from TRPV1 null mice. (**A**) Western blots of SC lysates probed for pPKA, pPI3K, and pPKC. (**B**) Expression levels of pERK, pp38, pJNK, and pAKT. (**A**) Western blots of SC lysates probed for pCREB, Nav1.7, and Nav1.8. α-tubulin was the internal control. *p < 0.05.

To further clarify the role of TRPV1 and associated pathways in inflammatory hyperalgesia, we examined the expression levels of the aforementioned proteins in the DRG of TRPV1^−/−^ mice. In contrast to TRPV1^+/+^, we found that potentiation of pPKA was alleviated in TRPV1^−/−^ mice DRG (Fig. 6A, 57.03 ± 10.32, *p* < 0.05, compared with control group, n = 6). Immunoreactive positive density of PI3K was reduced in TRPV1^−/−^ mice DRG (Fig. 6A, 62.72 ± 11.35, *p* < 0.05, compared with control group, n = 6). A similar result was also obtained for pPKC in TRPV1^−/−^ mice (Fig. 6A, 45.71 ± 5.35, *p* < 0.05, compared with control group, n = 6). Downstream signal molecules such as pERK, pp38, pJNK, and pAKT were further utilized for clarifying of TRPV1. The expression levels of pERK (Fig. 6B, 62.58 ± 8.37, *p* < 0.05, compared with control group, n = 6), pp38 (Fig. 6B, 66.63 ± 7.63, *p* < 0.05, compared with control group, n = 6), pJNK (Fig. 6B, 88.9 ± 3.3, *p* < 0.05, compared with control group, n = 6), and pAKT (Fig. 6B, 84.81 ± 8.53, *p* < 0.05, compared with control group, n = 6) were attenuated dramatically in TRPV1^−/−^ mice DRG. Furthermore, we verified that pCREB was decreased in TRPV1^−/−^ mice DRG (Fig. 6C, 50.6 ± 7.26, *p* < 0.05, compared with control group, n = 6). Next, we found that Nav1.7 (Fig. 6C, 61.7 ± 4.99, *p* < 0.05, compared with control group, n = 6) and Nav1.8 (Fig. 6C, 79.27 ± 5.18, *p* < 0.05, compared with control group, n = 6) were also alleviated in TRPV1^−/−^ mice DRG. Similar phenomena were also observed in SC lysates from TRPV1^−/−^ mice (Fig. 7). These results suggest that deletion of TRPV1 significantly reduced the upregulation of nociceptive associated signal mechanisms and may contribute to inflammatory hyperalgesia.

## Discussion

Our results demonstrated that ipsilateral and contralateral acupuncture has similar analgesic effect in CFA-induced inflammatory pain. Electrophysiological results also indicated that Nav currents increased in CFA-induced inflammatory pain and further were attenuated by EA. Administration of TRPV1 agonist could significantly increase Nav sodium current. Western blotting analysis revealed that the expression levels of proteins in TRPV1 and associated signaling pathways were attenuated in the DRG of both EA treatment groups. Similarly, in the central SCDH, the expression of TRPV1 increased following CFA injections and significantly decreased following EA at either ipsilateral or contralateral sites. The data from western blotting experiments supported the data from behavioral experiments, which indicated that the efficacy of contralateral acupuncture was similar to that of ipsilateral acupuncture.

There were many theories about the mechanisms underlying the analgesic effect of EA or MA, including the gate control and the endogenous opiates theories. The mechanism underlying the analgesic effect of contralateral EA is not yet clear. A study indicates that spinal μ-opioid receptor plays a critical role in contralateral EA analgesic mechanism^30^. The authors performed EA at both ST36 and SP9 before carrageenan injection as a pretreatment. This design was not compatible with clinical use, but the researchers found a possible analgesic mechanism at the spinal level. Different acupoints are used to treat different diseases in the clinic, although some people consider that acupoints like LI4 and ST36 belong to “pain control.” In a study on contralateral acupoints analgesia, it was SI6 and not LI4 that provided pain relief in the contralateral hindpaw^28^. There is also a study that reported that many acupoints to have a similar analgesic effect in knee arthritis in mice^37^. Accordingly, descending regulation of opioid concept in peripheral level is not enough to completely explain the analgesic effect.

Recent study indicated that the amount of TRPV1 is higher in acupoints than it is in tissue along the meridian and nonacupoints^38^. They also concluded that the expression of TRPV1 was increased after EA stimulation. This is crucial for mediating the transduction of EA signals to the CNS^38^. From an anatomic point of view, an acupoint such as ST36 is TRPV1 rich, and this may be the reason why some acupoints are used for pain control^3^,^39^. Gao *et al.* reported that acupuncture at ST36 acupoint could significantly increase gastric motility amplitude in rats with atropine-induced gastric inhibition by 1-3 Hz frequency^40^. EA, but not sham control, can reliably control body weight by increasing TRPV1 in DRG and SC^41^. In this experiment, both ipsilateral and contralateral low frequency electric acupuncture had analgesic effects. Interestingly, there is a study about thermal and mechanical allodynia in a CCI (chronic construction incision) mouse model that used contralateral TENS instead of EA treatment. There was no statistical change in behavior following low frequency TENS (2 Hz)^29^. Only high frequency or high/low frequency TENS could alter neurotransmitters and improve mechanical pain. Low frequency stimulation can reduce cold allodynia in chronic nerve injury through spinal adrenergic and serotonergic receptors^37^,^42^. Therefore, EA and TENS may achieve analgesia in different conditions and using different pathways. Accordingly, we suggest that TRPV1 is abundant in the nerve endings of the DRG near ST36 acupoint that is responding for EA analgesia.

In a previous study, the expression of TRPV1 in DRG increased after inducing peripheral inflammation and decreased after EA application^2^. Coexpression of pPKCɛ and TPRV1 revealed in inflammatory state, which may be due to TPRV1 phosphorylation induced by activated pPKC^43^. TRPV1 and pPKCɛ are also reported for thermal pain^44^. Furthermore, PKC was coexpressed with TRPV1 in DRG under thermal stimuli^45^. There are fewer studies about pPKCɛ in the spinal level, but it is known that PKC may relieve inflammatory pain by modulating endogenous opioids such as the ouabain-like substance (OLS) via affecting c-Fos in the dorsal horn^46^. TRPV1 located in spinal cord should be essential in thermal and mechanical hyperalgesia^47^. Similar patterns were also obtained in pPKA and pPI3K, suggesting its crucial role in inflammatory pain.

The expression of pERK is also important for inflammation, it reacts rapidly in DRG and dorsal horn of spinal cord^48^,^49^,^50^. Researchers found that regulation of TRPV1 in DRG may be modulated by the Ras–MEK–ERK pathway in inflammation^51^,^52^. At the spinal level, ERK is active by inflammation and is essential for hyperalgesia^53^. EA can reduce inflammatory pain via modulating ERK, and the mechanism may be inhibiting COX2 and CREB-NK-1 in the dorsal horn^54^. Auricular electroacupuncture can reduce epilepsy by altering pPKC and pERK signaling pathways in kainic acid-treated rats^55^. Signaling pathways of pPKC and pERK were also reported to be involved in neuronal mechsanotransduction^56^. Nociceptive Nav1.7 and Nav1.8 sodium channels overexpression were also reduced by EA and genetic TRPV1 manipulation suggesting a crucial role in inflammatory pain. Our results showed reduction in pPKA, pPI3K, pPKC, and related molecules in the DRG and SC, and these results corresponded to the behavioral observations. The results of this experiment are compatible with previous studies. Both ipsilateral and contralateral EA can reduce nociceptive signaling in DRG and SCDH.

## Conclusion

**Figure 8:**
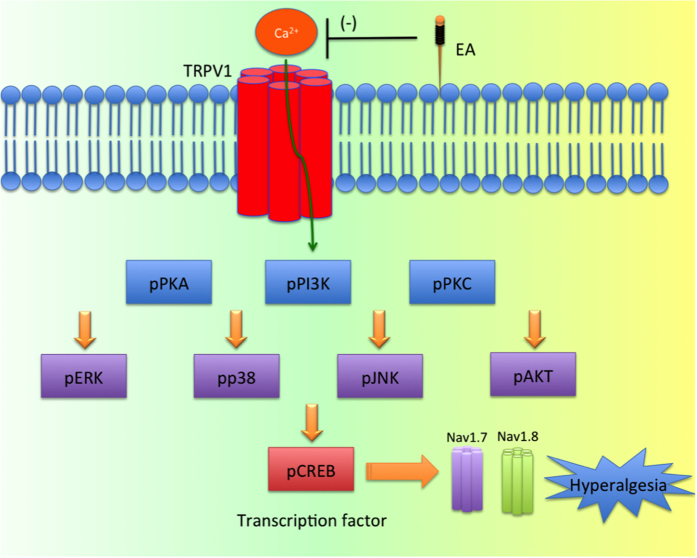
Schematic illustration of possible mechanisms in EA-mediated analgesia of CFA-induced inflammatory pain. Summary diagram of how TRPV1 is crucial for inflammatory pain and related mechanisms. Our results show that TRPV1 acts as a receptor in inflammatory pain. Activation of TRPV1 increases the expression of pPKA, pPI3K, pPKC. Furthermore, pERK, pp38, pJNK, pAKT, and pCREB were also increased. Moreover, nociceptive Navs were increased for pain conduction. Aforementioned molecules could be attenuated in TRPV1^−/−^ mice.

This study indicated a similar analgesic effect between ipsilateral and contralateral EA treatment on day 4 after inducing inflammatory pain in mice, and this effect was observed to become rapid after EA. Repeated EA treatment can further reduce pain but may reach a therapeutic plateau. Overexpression of TRPV1 and associated signaling pathways in both the DRG and SC were attenuated in TRPV1^−/−^ and EA-treated mice, and there was no significant difference between two EA treatments. This study provides a possible signaling mechanism of TRPV1 and relevant molecules (Fig. 8)

## Additional Information

**How to cite this article**: Lu, K.-W. *et al.* Probing the Effects and Mechanisms of Electroacupuncture at Ipsilateral or Contralateral ST36-ST37 Acupoints on CFA-induced Inflammatory Pain. *Sci. Rep.* **6**, 22123; doi: 10.1038/srep22123 (2016).
